# Activity-Dependent Release of Adenosine: A Critical Re-Evaluation of Mechanism

**DOI:** 10.2174/157015908787386087

**Published:** 2008-12

**Authors:** Mark Wall, Nicholas Dale

**Affiliations:** The Neuroscience Research Group, Department of Biological Sciences, University of Warwick, Coventry, CV4 7AL, UK

## Abstract

Adenosine is perhaps the most important and universal modulator in the brain. The current consensus is that it is primarily produced in the extracellular space from the breakdown of previously released ATP. It is also accepted that it can be released directly, as adenosine, during pathological events primarily by equilibrative transport. Nevertheless, there is a growing realization that adenosine can be rapidly released from the nervous system in a manner that is dependent upon the activity of neurons. We consider three competing classes of mechanism that could explain neuronal activity dependent adenosine release (exocytosis of ATP followed by extracellular conversion to adenosine; exocytotic release of an unspecified transmitter followed by direct non-exocytotic adenosine release from an interposed cell; and direct exocytotic release of adenosine) and outline discriminatory experimental tests to decide between them. We review several examples of activity dependent adenosine release and explore their underlying mechanisms where these are known. We discuss the limits of current experimental techniques in definitively discriminating between the competing models of release, and identify key areas where technologies need to advance to enable definitive discriminatory tests. Nevertheless, within the current limits, we conclude that there is evidence for a mechanism that strongly resembles direct exocytosis of adenosine underlying at least some examples of neuronal activity dependent adenosine release.

## INTRODUCTION

Adenosine is one of the most important modulators in the brain. Originally discovered as a powerful vasodilator [21], adenosine acts as: an endogenous somnogen [3]; a retaliatory neuroprotective analyte during metabolic stress [16,  54]; a modulator of pain pathways [56]; a modulator of spinal motor output [7,  18]; and in many regions of the brain a depressor of synaptic transmission.

### Receptors

Adenosine can act at 4 types of G-protein coupled receptors, respectively the A_1_, A_2A_, A_2B_ and A_3_ receptors (for comprehensive reviews see [26,  50]). A_1_ receptors are mostly inhibitory mainly coupling through the G_i/o_ type G proteins to inhibit adenylate cyclase. In some instances they can couple through G_q/11_ to stimulate phospholipase C. The A_2A_ and A_2B_ receptors are broadly similar to each other with the A_2A_ receptor being found in the central nervous system and the A_2B_ receptor in peripheral tissues. The A_2A_ receptor is largely excitatory coupling through G_s_ to stimulate adenylate cyclase. The A_3_ receptor is once again largely inhibitory and couples through G_i/o_ to inhibit adenylate cylase and stimulate phospholipase C.

### Mechanisms of Adenosine Production

A common consensus implicit in the literature is that adenosine is not released *via* exocytosis. Instead there are two main pathways for its appearance and production in the extracellular space. Firstly it can arise from previously released ATP through the actions of several different enzyme families collectively called the ectonucleotidases, reviewed by [29,  53,  64-66]. These families comprise: the E-NTPDases (of which there are at least 4 members located in the plasma membrane and which have a variety of substrate specificities and convert ATP to either ADP or AMP); the E-NPPases (of which there are 6 putative members, 3 of which have been shown to be capable of converting ATP to AMP with one variant also able to produce adenosine from AMP); the ecto-alkaline phosphatases (which have extremely broad substrate specificities) and the ecto-5’-nucleotidase, which converts AMP to adenosine. There is no doubt that this is an important route of extracellular adenosine production. It has been well documented in for example spinal cord where ATP is produced during motor activity (see later). This route is also important for the adenosine tone that is present within the brain, which arises from prior release of ATP from astrocytes [49]. 

However adenosine can also be released directly. This direct release seems to occur during pathologies such as oxygen-glucose deprivation (ischemia, [28]) and during hypercapnic acidosis in spinal cord [48]. The mechanism of release is uncertain, but is assumed to involve transporters. However there is conflicting evidence: for the most part the equilibrative transporters apparently act to remove adenosine from the extracellular space –their inhibition causes a rise in extracellular adenosine [19,  28]. This inward concentration gradient for adenosine may occur because adenosine kinase, a cytoplasmic enzyme, keeps the free intracellular adenosine concentration very low [5]. When this enzyme is inhibited, either pharmacologically [28,  60] or for example during hypercapnia in spinal cord [48], adenosine can be released *via* the equilibrative transporters.

There is also growing evidence (reviewed below) that adenosine can be released, under non-pathological conditions, by the activation of neurones by brief trains of action potentials. This potentially important route for adenosine release links neural activity (action potentials) with a mechanism to feedback and inhibit the activated pathway (*via* A_1_ receptor activation) and the possibility of inhibiting neighbouring pathways, depending on the extent of adenosine diffusion. It is also possible that the released adenosine can produce changes in blood vessel diameter [41] and thus link neural activity with blood flow, although several other mediators released from astrocytes have also been proposed [44,  59,  68]. Although the exact mechanism of how the adenosine is released remains unclear, there are hints that adenosine could be directly released by exocytosis. Below we outline some simple schemes that could account for activity-dependent adenosine release, discuss the way these schemes could be experimentally distinguished and summarise data from a number of different studies. 

#### Activity-Dependent Adenosine Release –Possible Mechanisms

Around 75% of energy expenditure in the brain is devoted to signalling [2]. One possible reason for activity-dependent adenosine release could be depletion of ATP levels during neural signalling. This depletion would however have to result in elevated levels of cytoplasmic adenosine to allow subsequent transport to the extracellular space. This may be significant during more global activity in the brain such as epileptic seizures, but seems an unlikely explanation for adenosine release that can be seen following brief activation of signalling (on the order of seconds) in small populations of neurons. Furthermore such a metabolic source of adenosine would be released in a Ca^2+^-independent manner and by a transport dependent process. As there is evidence for transport-independent and Ca^2+^-dependent mechanisms of adenosine release (see below), further mechanisms of activity-dependent release of adenosine must be considered.

We therefore consider three further simple schemes to provide potential mechanisms for the activity-dependent release of adenosine (Fig. (**1**)). All these schemes require exocytosis, as it is the simplest way of linking activity with release, and thus the release of adenosine in all cases will exhibit both calcium and action potential dependence. However only in case 3 will adenosine be packaged in vesicles and directly released by exocytosis. We shall consider these three schemes in detail and outline the critical experimental tests required to distinguish between them.

## SCHEME 1 (EXTRACELLULAR METABOLISM OF ATP) 

Under this scheme ATP, released by exocytosis, is broken down in the extracellular space by ectoATPases and nucleotidases to produce adenosine. This scheme includes the direct exocytosis of ATP from neurones and also exocytosis of ATP from glia in response to a transmitter, for example glutamate, released from neurones. Rapid ATP release will be followed by the slower appearance of adenosine in the extracellular space. The reason for this slower dynamic of adenosine production is that ATP catabolism is a multienzyme process with the final step, conversion of AMP to adenosine, being subject to feed-forward inhibition by ATP and ADP [32].

A critical test of this scheme is to examine the sensitivity of adenosine production to blockers of the ectoATPases (such as ARL 67156) and the ecto- 5’-nucleotidase (such as α,β-methylene ADP). These should diminish the production of adenosine and hence reduce its effects; blockade of the ectoATPase may also enhance any direct effects of ATP mediated through P2 receptors. Ideally, direct measurement of extracellular ATP *via* luciferin-luciferase chemiluminescence (for example [46]) or with biosensors [37] and adenosine with biosensors [19,  36] or fast cyclic voltammetry [13,  58] should be attempted. With these techniques, it may be possible to directly demonstrate the characteristic delays in adenosine production with respect to neural activity that betokens extracellular conversion of ATP and the intervening metabolites [19,  20,  32].

Thus the absence of an effect of blockade of extracellular ATP catabolism on adenosine production and the appearance of adenosine in the absence of any ATP release would eliminate this scheme from consideration, subject to the caveats outlined below.

## SCHEME 2 (INTERPOSED TRANSMITTER)

Under the second scheme, an unspecified neurotransmitter would be released (by exocytosis) to activate an interposed cell (either a neuron or astrocyte) to produce adenosine release (by some currently unclear mechanism).

The discriminatory test would be to block the receptors of the interposed transmitter (with selective antagonists) – these should diminish adenosine release. The difficulty is that the identity of the interposed transmitter will be unknown; therefore even use of an exhaustive battery of antagonists may not be conclusive if these all provide negative results. There are many examples where activation of glutamate receptors has been shown to release adenosine (for review see [35]). Determining whether adenosine release is sensitive to glutamate receptor blockade is thus an important test, however other types of transmitter should also be examined including the cannabinoids, which can induce changes in intracellular Ca^2+^ in neurons and astrocytes [22]. If the interposed cell were to be an astrocyte, then it is vital to directly image astrocytic signalling (*via* intracellular Ca^2+^) to demonstrate the efficacy or otherwise of the antagonist regimen in preventing astrocytic activation.

An alternative and more general way of testing this scheme is to try and inactivate the putative interposed cell. If this is a neuron, then massive activation of GABA_A_receptors will shunt the membrane and hence the excitatory effects of the putative interposed transmitter, without greatly diminishing release of the transmitter itself. A difficulty arises if the interposed cell is an astrocyte as these may not possess GABA_A_ receptors. Some authors have used fluorocitrate and fluoroacetate to alter astrocytic function [39]. We have concerns as to the specificity of such treatments and whether they are adequate tests of astrocytic involvement. Other ways to test the involvement of interposed astrocytes that may be of utility would be their selective loading with Ca^2+^ chelators (possible in brain slices derived from young rodents [17]) or the use of genetically altered mice where astrocytic signalling has been selectively disrupted [49].

However we conclude that is very hard to completely eliminate this scheme as an explanation of adenosine release –at any point a positive result will demonstrate that this scheme applies, e.g. sensitivity of adenosine release to glutamate receptor antagonism, but a series of negative results to all of these tests cannot completely exclude this explanation either.

## SCHEME 3 (DIRECT EXOCYTOSIS OF ADENOSINE)

The final mechanism is the direct release of adenosine by exocytosis. This implies the loading of adenosine into synaptic vesicles –perhaps hard to achieve if cytoplasmic adenosine levels are very low, which can be the case for cells in which adenosine kinase is strongly expressed. However a recent report suggests that adenosine kinase is largely absent from hippocampal neurons of adult rat supporting the plausibility of vesicular loading [57].

Under this scheme it is necessary to show the release of adenosine in the absence of the release of ATP as well as insensitivity to blockers of ATP catabolism. However conclusive demonstration of adenosine exocytosis is difficult. One obvious reason for this is that adenosine does not act through a ligand-gated channel thus the possibility, which would definitively and easily demonstrate this scheme, of observing adenosinergic miniature postsynaptic currents or potentials, does not exist.

An excellent, but difficult, test is to block exocytosis from the cell type hypothesized to release adenosine –this may be possible with genetically altered rodent models (for example [63]), however these data would not necessarily exclude involvement of an interposed cell (scheme 2). Demonstration of the presence of adenosine in synaptic vesicles would provide supporting evidence for this hypothesis. Ultimately however, definitive evidence will require demonstration of adenosine release *via* exocytosis from isolated cells (either acutely or in culture). Further developments in adenosine sensing technologies may make this test possible.

## EXAMPLES OF ACTIVITY DEPENDENT ADENOSINE RELEASE

There are a number of regions of the central nervous system where activity-dependent adenosine release has been described. We will examine them and discuss the evidence for which scheme may underlie the release.

### *Xenopus* Spinal Motor Pattern Generation

ATP and adenosine modulate spinal motor pattern generation in *Xenopus* embryos. ATP is excitatory acting through P2Y receptors to inhibit voltage gated K^+^ channels [9,  18]. Adenosine depresses motor activity, and acts by inhibiting voltage-gated Ca^2+^ channels [8,  18]. The dynamic balance between these modulators changes with time during swimming activity and results in a gradual slowing and spontaneous termination of the motor pattern. This system involves the release and production of both ATP and adenosine –their postsynaptic effects can be demonstrated *via* the use of antagonists. Direct demonstration of ATP release from isolated neurons [10], and from the spinal cord during locomotion has been achieved [37]. Adenosine production during swimming has also been demonstrated [19,  36]. This has the predicted slow dynamic arising from extracellular catabolism of ATP [19,  20,  36]. Thus this is a clear example of adenosine release arising *via* scheme 1.

### Hippocampal CA1 Synapses

Glutamatergic synapses onto CA1 pyramidal neurones are modulated by the activation of A_1_ receptors leading to presynaptic inhibition [24,  55]. Mitchell *et al.* (1993) [42] provided the first evidence for a form of activity-dependent adenosine release at these synapses. They showed that the activation of an excitatory pathway onto CA1 neurones leads to the transient depression of fEPSPs evoked in another independent pathway (Fig. (**2**)). This inhibition was produced by the release of adenosine (presumably from a component within or activated by the stimulated pathway) and the subsequent activation of presynaptic A_1_ receptors (as the inhibition could be blocked by the adenosine receptor antagonist theophylline). Although the depression was seen with a single stimulus it was much more pronounced with a train of stimuli (16-23, at 100 Hz) presumably as more adenosine is released [42]. The degree of inhibition was also increased if the extracellular breakdown of adenosine was reduced with an adenosine deaminase inhibitor (EHNA) and thus presumably greater concentrations of adenosine can diffuse to the depressed pathway [42]. Similar results have also been observed using organotypic slices, where the short-term depression of a single CA1 pathway (with a 10 Hz, 5 s stimulation) was partly reversed by blocking A_1_ receptors [6]. Thus the adenosine released can inhibit both the stimulated pathway and diffuse to inhibit neighbouring pathways. Functionally, adenosine release occurring during high frequency stimulation can block the consolidation of LTP [1,  14]. Activity-dependent adenosine release is also seen during seizures generated in the hippocampus and acts to terminate the seizures [25].

What is the mechanism of this activity-dependent adenosine release? Addition of a 5’-ectonucleotidase inhibitor (200-500 µM α,β  methylene-ADP) had no effect on the degree of synaptic depression [6,  42] suggesting that the adenosine does not arise from the extracellular metabolism of ATP (eliminating scheme 1). The adenosine release persisted in 10 µM dipyridamole [6] suggesting that it may not arise from cytoplasmic translocation *via* a carrier protein, nevertheless this possibility is not completely excluded as a dipyridamole-insensitive carrier could still be involved. There is currently no data on whether the release of an interposed transmitter is required for adenosine release or indeed whether the adenosine release involves exocytosis. However, Manzoni *et al.* (1994) [38] have shown that NMDA receptor activation causes the release of adenosine and subsequent inhibition of CA1 synapses *via* A_1_ receptor activation. This adenosine release persisted in the presence of α,β methylene ADP (500 µM) and thus does not appear to involve the breakdown of extracellular ATP. Slice superfusion experiments suggest that this adenosine release is Ca^2+^ dependent [15] and thus may involve exocytosis. The obvious question is: does activity-dependent release of adenosine produced by synaptic stimulation require NMDA receptor activation (implying a scheme 2 type mechanism)? This currently remains unknown but would be relatively easy to answer by stimulating CA1 synapses with trains, with and without an NMDA receptor antagonist. 

What is the source of the adenosine? Brundege and Dunwiddie (1996) [11] showed that loading CA1 pyramidal neurones with adenosine (*via* a patch pipette) led to adenosine release and the presynaptic inhibition of glutamatergic synaptic transmission on to the cell. However loading the neurones with enzyme inhibitors and nucleotides had no effect on the degree of synaptic transmission suggesting that pyramidal cells do not readily form adenosine from endogenous sources [12]. Manzoni *et al.* (1994) [38] suggested that the NMDA receptor mediated adenosine release could be *via* inhibitory interneurones as it was reduced by enkephalin, which selectively inhibits interneurones. 

### The Calyx of Held

The Calyx of Held is a giant nerve terminal in the auditory brainstem forming an axo-somatic glutamatergic synapse onto a principal cell in the medial nucleus of the trapezoid body (MNTB). Transmission at this synapse is depressed by the activation of presynaptic adenosine A_1_ receptors [4] an effect that declines during postnatal development [33]. Kimura *et al.* (2003) [33] showed, with an A_1_ receptor antagonist (8 CPT), that there is no tone of adenosine continually activating the A_1_ receptors. However during high frequency trains (10 Hz), blocking A_1_ receptors relieved a proportion of the synaptic depression, suggesting that adenosine release occurred during the train (Fig. (**3**)) [33]. By comparing the degree of inhibition relieved by 8 CPT (~13 %) and the adenosine concentration-response curve it was possible to estimate the concentration of adenosine released, which reached ~ 5 µM [33]. 

What is the mechanism for this activity-dependent adenosine release? Blocking extracellular ATP metabolism (with the ectoATPase inhibitor ARL 67156, 50 µM) had no effect suggesting the adenosine does not arise from the extracellular breakdown of ATP (eliminating scheme 1, [62]). This was supported by the inability of the luciferin-luciferase assay to detect any ATP release following a 200 Hz train [62]. However there is currently no information on whether the adenosine release requires exocytosis and whether the release of an interposed transmitter is required. The most likely source of the adenosine is the excitatory terminals (which release glutamate and express the A_1 _receptors) and thus the adenosine acts to depress excitatory synaptic transmission (and presumably its own release) *via* autoreceptors [62]. 

### Parallel Fibre-Purkinje Cell Synapses in the Cerebellum 

Glutamatergic parallel fibre to Purkinje cell synapses are inhibited by the activation of presynaptic adenosine A_1_ receptors [34,  52]. Adenosine biosensors [36] placed on the surface of the molecular layer of transverse slices (above the parallel fibre tracts) can detect adenosine release following trains (5-80 Hz) of stimuli (Fig. (**4**)) [61]. The amount of adenosine released was increased by increasing the number of stimuli and by the higher frequencies of stimulation. Sufficient adenosine was released to inhibit parallel fibre-Purkinje cell synaptic transmission, an effect blocked by A_1_ receptor antagonists [61].

What is the mechanism of adenosine release? As with the hippocampus and Calyx of Held, the amount of adenosine released was not reduced by blocking extracellular ATP breakdown (with Evans Blue, ARL 67156 or α, β-methylene ADP at 100 µM) [61]. Furthermore ATP biosensors (detection limit ~ 60 nM, [37]) placed either in the slice or in the same position as the adenosine biosensor did not detect ATP release following trains of stimuli [61]. Release of ATP by local electroporation [c.f. 30] led to rapid adenosine production (*via* extracellular metabolism) but the proportion of ATP broken down to adenosine was relatively small (~20 %) suggesting that complete and rapid ATP breakdown was extremely unlikely [61]. Thus it appears that the source of adenosine is not extracellular ATP eliminating scheme 1. The adenosine release was reversibly blocked by tetrodotoxin (action potential dependent) and was also Ca^2+^ dependent [61]. Thus the release mechanism appears to involve exocytosis and thus probably occurs either by scheme 2 (interposed transmitter release) or by scheme 3 (direct release of adenosine by exocytosis). Adenosine release was not reduced by blocking a number of transmitter receptors (including glutamate, noradrenaline and 5 HT) and was not reduced by shunting neurones and glia by activating GABA_A_ receptors with muscimol [61]. Furthermore release was not reduced by blocking adenosine transport *via* ENT1 and ENT2 (with NBTI/dipyridamole). Thus it appears unlikely that the adenosine release occurs by scheme 2. The simplest explanation is that the release mechanism is direct exocytosis of adenosine.

What is the source of the released adenosine? The position of the stimulating and recording electrodes, along a beam of parallel fibres, are ideal to stimulate and record release from parallel fibres. Furthermore, activation of presynaptic receptors on parallel fibre terminals (GABA_B _mGluR4 and A_1 _receptors) causes a reversible reduction in adenosine release [61]. The restricted location of mGluR4 receptors in the cerebellum (only present on parallel fibre terminals [40]), strongly suggests that adenosine release occurs from the terminals of parallel fibres. Block of A_1_ receptors (with 8 CPT) markedly increased the amount of adenosine released suggesting that the A_1_ receptor acts as an autoreceptor, controlling the amount of adenosine and glutamate released [61]. 

## OTHER SYNAPSES

In the rat hypothalamic supraoptic nucleus, blocking A_1_ receptors had no effect at a low frequency synaptic stimulation (0.05-0.5 Hz) but at 1 Hz, the time-dependent depression of inhibitory and excitatory transmission was prevented by A_1_ receptor block [47]. Thus it appears that activity-dependent adenosine release occurs during trains of activity in a similar fashion to the other synapses. 

### Limitations of Studies

There are at least three experimental limitations that prevent the full elucidation of the mechanisms underlying activity-dependent adenosine release:

### Low Efficacy of the Inhibitors of Extracellular ATP Catabolism

Critical for the evaluation of scheme 1, is to test whether adenosine arises from the extracellular breakdown of ATP by using inhibitors of the ectoATPases and ecto-5’-nucleotidase. This is fine if the inhibitor has a positive effect (reducing the amount of adenosine released). However a lack of effect, may be a false negative as the agents may not have effectively blocked the breakdown of ATP. This is plausible, as the widely used NTPDase inhibitor, ARL 67156, has relatively low efficacy and is a competitive antagonist. High concentrations of ATP will overcome its relatively weak blocking action [28,  43,  61]. In cerebellum, the 5’-nucleotidase inhibitor α,β-methylene ADP appears the most effective agent in preventing the production of adenosine [61] but its action is competitive and thus will be overcome by high levels of substrate. By contrast α,β-methylene ADP is ineffective at blocking the ecto-5’-nucleotidase in *Xenopus* and acts instead as an agonist at a P2Y1 receptor [9]. Indeed several P2 receptor antagonists such as suramin, PPADS, NF449, NF279 and RB-2 are effective blockers of at least some E-NTPDases [31,  45,  68], complicating interpretations over the use of these compounds.

There are convenient methods to analyze the extracellular catabolism of ATP *in situ* and in brain slices [9,  28,  60,  61]. These allow determination of the efficacy of putative inhibitors of the ectoATPases and ecto-5’-nucleotidase and we strongly recommend their use to give greater credibility to the interpretations resulting from the use of these compounds.

Many studies only measure adenosine release indirectly *via* the inhibition of synaptic transmission by the activation of presynaptic A_1 _receptors. Because the A_1_ receptors have a very high affinity for adenosine (EC_50_ 600-750 nM for depressing CA1 EPSPs, [23]), it is probable that only a small proportion of the extracellular ATP has to be broken down to produce significant A_1_ receptor activation. Thus if the inhibitors are only partially effective, enough adenosine may still be produced to cause synaptic inhibition. Although this problem can be reduced by combining the inhibition of ATP breakdown with other methods such as biosensors [60,  61] and luciferin-luciferase [62] to detect ATP release, there always remains the doubt that if no ATP is detected it is because of extremely rapid breakdown that has not been prevented by the inhibitor. Thus future studies require either the development of new more effective NTPDase inhibitors (for example see [43]) or the use of transgenic technology to prevent extracellular ATP breakdown.

## INDIRECT MEASUREMENTS OF ADENOSINE RELEASE 

Most of the studies of activity-dependent adenosine release, indirectly measure the adenosine released by the activation of A_1_ receptors and the subsequent inhibition of synaptic transmission. There are two major problems with this method: firstly any procedure that modulates adenosine release but also changes synaptic transmission cannot be examined. For example procedures such as removing extracellular Ca^2+^ or blocking action potentials (with tetrodotoxin) cannot be used as they will also block synaptic transmission and thus the influence of adenosine on transmission can no longer be measured. Secondly, the quantification of the amount of adenosine is difficult as the degree of synaptic inhibition is not always linearly related to the amount of adenosine present (at the bottom of the concentration curve and at the top where receptors are saturated). This problem can be avoided by combining measurements of synaptic inhibition with a direct real-time measure of adenosine release using for example a biosensor [27,  61].

## EXCLUSION OF AN INTERPOSED TRANSMITTER

It is difficult to distinguish whether adenosine is directly released by exocytosis or whether there is an interposed transmitter (schemes 2 and 3). This is because there are a large number of possible transmitter candidates and it is difficult to test them all. Wall & Dale (2007) [61] used the shunting of neurones and glia with the GABA_A_ receptor agonist muscimol to exclude release from interposed neurones and glia. Although Purkinje cells were effectively shunted, the effects on glia remain unclear although they do express GABA_A_ receptors [51]. Future strategies could include the use of transgenic mice, where exocytosis from glia is blocked (dominant-negative SNARE, [49]) or transmission at specific synapses is blocked (for example by selective tetanus toxin expression, [62]) or the measuring of adenosine release from cultures of specific cell types.

## CONCLUSIONS

There is growing evidence that activity-dependent adenosine release occurs in several different brain areas and thus is perhaps a common feature of many central neural circuits. In all of the reports, a train of electrical activity (usually in glutamatergic neurones), releases adenosine which feeds back to inhibit transmitter release in the stimulated pathway. In many cases, although the adenosine does not arise from extracellular ATP breakdown (but see the caveats above) it is still unclear where and how the adenosine is released. In the cerebellum, where the story is most complete [61], the simplest mechanism that accounts its release is the direct exocytosis of adenosine from parallel fibres, although future experiments are required to prove this definitively. The use of biosensors in the cerebellum has allowed much greater insight into the properties of adenosine release compared to studies which only monitored adenosine release indirectly (*via* synaptic inhibition). For example, cerebellar adenosine release is modulated by the activation of presynaptic receptors such as GABA_B_ and mGluR4 and adenosine itself can feedback to inhibit its own release (*via* A_1_ receptor activation). Thus the adenosine release appears more like conventional transmitter release than perhaps expected and in many ways can be treated as a slow postsynaptic potential. Furthermore, the complexity of adenosinergic signalling is enhanced, as the amount of adenosine released will not only depend on the stimulus but also can be potentially modulated by several feedback mechanisms. Thus the full understanding of how adenosine is released and its effects on neural circuit behaviour will require the combination of electrophysiology, biosensor technology, computational studies and the development of new tools, for example effective NTPDase blockers and transgenic technology.

## Figures and Tables

**Fig (1). Possible mechanisms for activity-dependent adenosine release.**
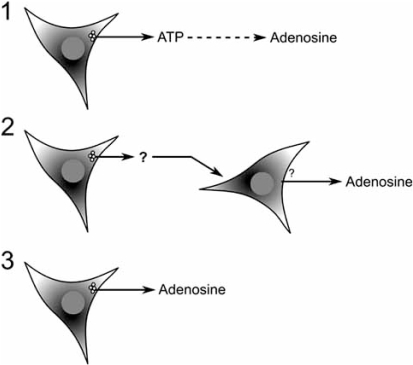
1) ATP is released by exocytosis (either from a neuron or glial cell) and subsequently metabolised in the extracellular space to produce adenosine. 2) An unspecified interposed transmitter (?) is released by exocytosis to act on a downstream cell causing the release of adenosine by an unknown mechanism (?). 3) Direct exocytotic release of vesicular adenosine

**Fig (2). Transient inhibition of hippocampal CA1 field EPSPs by conditioning stimulation.**
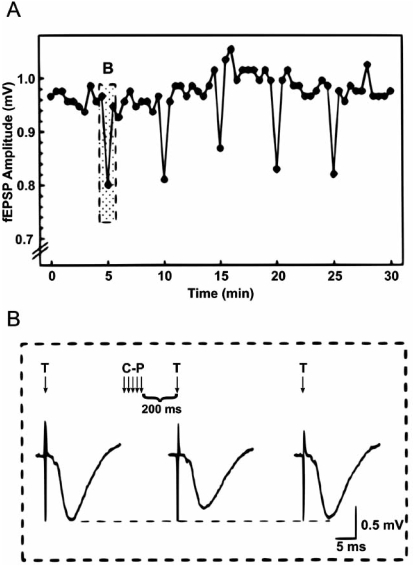
Field excitatory postsynaptic potentials (fEPSPs) were measured in the apical dendrites of CA1 and were stimulated by an electrode placed in the stratum radiatum of CA2. A conditioning electrode was placed in the stratum radiatum of CA1 near the subiculum. A) Amplitudes of fEPSPs evoked every 30 seconds. At 5 minute intervals, a conditioning train consisting of 5 pulses was applied ending 200 ms prior to the fEPSP. B) fEPSPs from the shaded area of A. Responses are shown to test stimulation alone (T) and 200 msec after the conditioning pulses (C-P T). fEPSPs preceded by the conditioning pulses were reduced 15-20 % relative to the baseline. Taken with permission from [42].

**Fig (3). Presynaptic inhibition by endogenous adenosine is increased during high frequency stimulation at the Calyx of Held:**
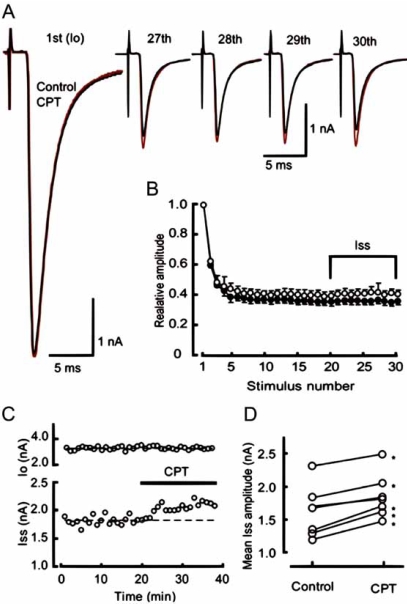
Excitatory postsynaptic currents (EPSCs) were recorded from MNTB principal neurons in P5-7 rats in response to stimulation of input fibres. A) Averaged EPSCs during a train of 30 stimuli at 10 Hz. The first EPSC (*I_0_*) and the 27th-30th EPSCs, before (black) and during (red) application of CPT (0.5 µM), are shown (superimposed). B) Synaptic depression during 10 Hz stimulation. EPSCs during a train are normalized in amplitude to the first EPSC. Mean amplitudes and S.E.M.s of EPSCs (from five cells with significant increase in mean amplitude of 20th- 30th EPSCs (*I_ss_*) after CPT application, D) during 10 Hz stimulation are plotted, before (●) and during (○) CPT application. C, Time plot of I_0_ and *I_ss_* in a cell. Bath application of CPT (bar) increased *I_ss_* with no effect on *I_0_*. Mean amplitude of Iss before CPT application is indicated by a dashed line. D) mean amplitudes of *I_ss_* before and after application of CPT in seven cells. Difference was statistically significant (*P < 0.05) in five cells shown in B, but insignificant in two other cells. Taken with permission from [33].

**Fig (4). Properties of adenosine release at cerebellar parallel fibre-Purkinje cell synapses.**
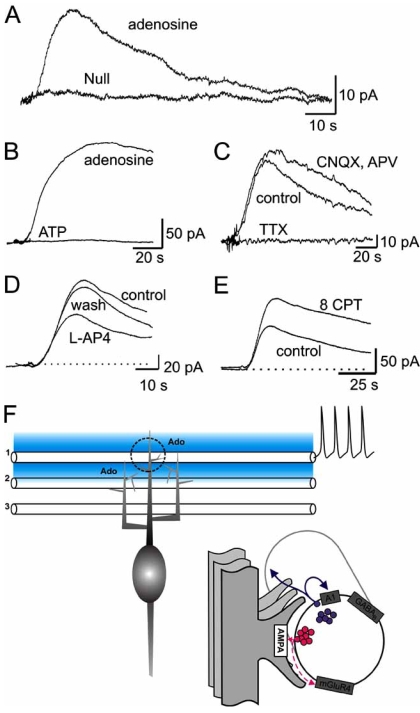
Biosensor recordings were made from transverse cerebellar slices from P21-28 rats. Biosensors were laid on the surface of the molecular layer and adenosine release was evoked with a stimulating electrode placed on the same beam of parallel fibres. A) Superimposed current traces from adenosine and null biosensors following electrical stimulation (20 Hz, 5 s). The lack of signal on the null sensor indicates the current on the adenosine biosensor is due to purine detection and is not non-specific. B) Superimposed traces from an adenosine biosensor placed on the surface of the molecular layer and an ATP biosensor placed within the molecular layer (under the adenosine sensor). Following stimulation, although adenosine was measured, no ATP could be detected. C) Superimposed current traces from an adenosine biosensor in control, CNQX (10 µM) and AP5 (50 µM, to block glutamate receptors) and TTX (0.5 µM, to block action potentials). The adenosine released by stimulation was blocked by TTX but was insensitive to the block of ionotropic glutamate receptors. D) Superimposed current traces from an adenosine biosensor in control and in the presence of the mGluR4 receptor agonist L-AP4 (50 µM). The amount of adenosine released following stimulation was reversibly reduced by the activation of mGluR4 receptors. E) Superimposed traces from an adenosine biosensor in control and in the presence of the A1 receptor antagonist 8CPT (1 µM). Block of A1 receptors markedly increased the amount of adenosine released. F) Current model of adenosine release. Trains of parallel fibre action potentials release adenosine (Ado, shaded area) which can diffuse between fibres. Inset, close-up of parallel fibre-Purkinje cell synapses (dotted area in A). Following an action potential train, active fibres release adenosine and glutamate. Adenosine feeds back to inhibit its own release and glutamate release, *via* A1 receptors. Adenosine may also diffuse to inhibit release from neighbouring inactive fibres (lateral inhibition). The release of adenosine is also inhibited by mGlu4R receptor activation (which could occur by feedback of released glutamate) and GABA_B_ receptor activation. A-E modified from [60].

**Table 1:** Summary Comparison of Activity-Dependent Adenosine Release in the Four Circumstances Discussed in the Text. The first three examples are candidates for direct activity-dependent release of adenosine. (*) In *Xenopus* activity dependent adenosine release occurs via extracellular conversion of ATP released as a result of self-sustained activity within the spinal motor network and the issues of Ca^2+-^ and action potential-dependence are implicit. (?) denotes that the dependence of mechanism respectively on Ca^2+^, TTX or interposed transmitter has not been tested.

	Trains of Stimuli Required for Release	Ca^2+^ Dependent	Action Potential Dependent (TTX)	Evidence for ATP Release	Evidence for an Interposed Transmitter
Hippocampal CA1 synapses	Yes^a^,^b^	?	?	No^a^,^b^	?
Calyx of Held MNTB	Yes^c^,^d^	?	?	No^d^	?
Cerebellar parallel fibre-Purkinje cell	Yes^e^	Yes^e^	Yes^e^	No^e^	No^e^
*Xenopus *spinal cord	Yes^f^	*	*	Yes^g^,^h^	

a [42]

b [6]

c [33]

d [61]

e [60]

f [18]

g [10]

h [37]
